# Determinants of Physical Activity and Sedentary Behavior in German Elementary School Physical Education Lessons

**DOI:** 10.3389/fspor.2020.00113

**Published:** 2020-09-15

**Authors:** David Jaitner, Michael Bergmann, Arvid Kuritz, Christoph Mall, Filip Mess

**Affiliations:** ^1^Department of Sports Science and Movement Pedagogy, Technical University of Braunschweig, Braunschweig, Germany; ^2^Chair for the Economics of Aging, Technical University of Munich, Munich, Germany; ^3^Munich Center for the Economics of Aging (MEA), Max Planck Institute for Social Law and Social Policy, Munich, Germany; ^4^Department of Sport Science, University of Konstanz, Konstanz, Germany; ^5^Department of Sport and Health Sciences, Technical University of Munich, Munich, Germany

**Keywords:** physical education, elementary school, Baden-Wuerttemberg (Germany), physical activity, accelerometer, moderate-to-vigorous physical activity (MVPA)

## Abstract

Physical activity (PA) in school physical education (PE) is a signature component of health promotion and health education. The study's aim was to explore PA levels and sedentary time in German elementary school PE lessons and relate them to selected personal and environmental PA determinants. Accelerometer measurements were collected from 328 students (47% male, mean age 8.7 ± 1.2 years) in 11 elementary schools in Baden-Wuerttemberg (Germany). PA levels and sedentary time were analyzed regarding gender, grade, body mass index, selected correlates of active living and health behaviors, as well as the PE teachers' PE education status. In line with previous research, the analyses of PA levels and sedentary time confirm gender and grade differences and highlight older girls as the less active group. Deviant weight status and parents' PA levels were found to be important determinants for PA levels and sedentary time of girls and offer starting points for intervention studies as well as gender-appropriate PE in elementary schools. Specialist PE teacher status proved to be a negative determinant of PA levels and sedentary time for boys and girls and should be investigated in further studies, especially regarding the didactic and methodological background.

## Introduction

Time is a recognized key factor in coordinating educational practice and a widely used variable in educational inquiry (Kakkori, 2013; Scheerens et al., 2013). In physical education (PE) research and sports pedagogy, time aspects are dominant especially in two research strands. First, teacher evaluation studies focus on physically skilled students and have systematically addressed the relationship between time and motor skill learning in school PE since the 1970's (Van der Mars, 2006; see Metzler, 1989 for initial studies). The associated core variable of these studies is academic learning time in PE (ALT-PE), widely operationalized as motor activity time at an appropriate level of task difficulty (Siedentop et al., 1982). Second, research considering the potential contribution of PE lessons to student health promotion and public health concentrates on physically active students and has been concerned with the relationship of time and physical activity (PA) in school PE since the 1980's (Trost, 2006; see Seliger et al., 1980; Callesen et al., 1983 for initial studies). Here, the associated benchmarks have been the proportions of PE time students spend in different PA levels and sedentary behaviour (SB). The primary focus has been on moderate-to-vigorous physical activities (MVPA), i.e., physical activities at the equivalent intensity between brisk walking and running/team games (Stratton, 1996; Fairclough and Stratton, 2006; Hollis et al., 2016, 2017). Recent studies complement objectively measured health benefits of light physical activities (LPA), i.e., physical activities at the equivalent intensity of slow walking (Ekelund et al., 2019), and reduced sedentary time, i.e., physical activities at the equivalent intensity of waking behavior in a reclining, seated, or lying posture requiring very low energy expenditure (De Rezende et al., 2014).

As life in modern industrial societies is characterized by noxious lifestyles and the existence of non-communicable chronic diseases (Kohl et al., 2012; Lee et al., 2012; Ding et al., 2017), the second research strand outlined above has significantly gained scientific weight and political support in recent decades (Sallis and McKenzie, 1991; Pate et al., 2006; Sallis et al., 2012). Besides unhealthy diet and legal intoxicants, insufficient PA and sedentariness rates are among the main risk factors (Tremblay et al., 2011; Durstine et al., 2013; González et al., 2017). As PA is credited with various prevention and health effects on the downside (Blair and Morris, 2009; Janssen and LeBlanc, 2010; Warburton and Bredin, 2017), school PE has received considerable relevance for public health: On the one hand, PE provides a regular contribution to health-promoting PA of children and adolsecents and at the same time can develop stable lifetime PA habits for the entire lifespan. Compulsory formal education reaches the full social spectrum of children and adolescents, especially those students who obtain little or no PA outside school PE. The early phase of life carries a particularly formative potential in itself (Malina, 2001; Morgan et al., 2007; Demetriou and Höner, 2012; Cardinal, 2016). On the other hand, students spend a considerable amount of their time awake at school (Craw, 2019), which has been further enhanced by the widespread introduction of full-day schools in many countries in recent years. On the one hand, this leads to a relatively simple possibility to broadly expose children and adolescents to health promotion and education (Naylor and McKay, 2009). On the other hand, it poses a risk of providing substantial contribution to sedentary time and behavior (Nettlefold et al., 2011; Egan et al., 2019).

Regarding PE research on time and PA, a substantial body of evidence has developed. Following established frameworks that explain factors of active living and health behaviors (Sallis et al., 2006; Trost, 2007), studies have explored students' physical activities in PE in various countries considering accompanied personal and environmental correlates (e.g., psychological, biological, sociodemographic, ecological, or cultural determinants) and comparing the results with national and transnational PA health recommendations. Most studies focus on PE in the United States (e.g., Simons-Morton et al., 1994; McKenzie et al., 1995, 2000, 2004; Nader, 2003; Scruggs et al., 2003; Surapiboonchai et al., 2012; Robinson et al., 2014; Harvey et al., 2016). Besides, there are studies in some European (e.g., Verstraete et al., 2007; Waring et al., 2007; Carreiro da Costa et al., 2008; Meyer et al., 2013; Fröberg et al., 2017) and a few Asian countries (e.g., Chow et al., 2008; Tanaka et al., 2018; Zimmo et al., 2020). Compatible analyses of German PE, however, have nearly not been introduced to international research yet (see Kobel et al., 2015 for an exception). Additionally, there is a limited body of studies describing, sorting, and categorizing time variables in German school PE in general. Considering the health significance of early life years as well as the relatively limited evidence base in Germany, the purpose of the present study was to assess PA levels in German elementary school PE lessons and by this contribute to national and international research on time and PA in PE.

### PA in International Elementary PE

International PE research on time and PA in school PE mainly addresses the direct contribution of PE lessons to students' health. In general, studies refer to an argumentation relating social urgencies (e.g., sedentary lifestyles, widespread diseases), to empirically proven prevention possibilities of PA (e.g., positive effects on health risk factors, contributions of PA in PE lessons to total PA per day/week), a globally recognized status of health in PE curricula, and so far unused opportunities in PE lessons (Sallis and McKenzie, 1991; Seefeldt, 1998; Sallis et al., 2012). An essential part of research emphasizes the health significance of early life years and focuses on elementary school PE lessons. Two reviews currently exist in the subject area of time and PA in elementary school PE (Fairclough and Stratton, 2006; Hollis et al., 2016). Fairclough and Stratton (2006) reviewed studies examining PA levels in elementary school PE published between 1980 and 2004. The narrative review included *n* = 44 studies with different study designs (i.e., cross-sectional, longitudinal, intervention). In classes taught under regular conditions, the mean lesson length was 33.7 min. Students were engaged in MVPA for 34.2% (12.6 min) of the available time and fell well-below the consulted recommendation of the U.S. Department of Health and Human Services (USDHHS) to be engaged in MVPA for 50% of the lesson on average. Regular PE lessons focusing on increased student MVPA led to higher intensities of PA and partly met the respective movement time recommendation. Subgroup analyses showed a trend of increasing PA levels with school grade (esp. between grades 3 and 5) and rather similar MVPA for boys and girls. Different PE curriculum areas were rarely explored and are therefore difficult to compare. The teacher's PE education status has proven to be a key variable in intervention programs enhancing PA in PE, favoring PE specialists and in-service classroom teachers over non-specialists, i.e., regular classroom teachers.

Hollis et al. (2016) systematically reviewed student MVPA levels during elementary school PE lessons in studies published between 2005 and 2014 (*n* = 13, seven thereof with sufficient data to be pooled into meta-analysis). Regarding the included studies, the percentage of PE time spent in MVPA ranged from 11.4 to 88.5%. Studies included in the meta-analysis revealed 44.8% of PE time in MVPA. Five of the 13 included studies exceeded the consulted recommendations of the U.S. Centre for Disease Control (CDC) and the U.K. Association of Physical Education (afPE) to be active in MVPA for at least 50% of the lesson. Moderator analyses found an explicit difference in the percentage of class time in MVPA between studies using observational measures (57.6%) and accelerometers (32.6%). Group differences for student (e.g., grade, gender), teacher (e.g., PE education status), and environmental factors (e.g., country) were not included in the meta-analysis. However, the atypical rate of specialist or qualified PE teachers in 8 of the 13 reviewed studies at least partially explains the increased mean percentage of MVPA per class and reinforces the PE teacher's education status as important moderator variable.

### Time and PA in German PE Research

Germany's general school system is subject to the cultural sovereignty of the individual federal states and comprises an elementary education in which all students are taught together for at least the first 4 years (age 6–10), followed by a structured secondary education in which students are typically separated in different school types according to their academic performance (i.e., general secondary school, comprehensive secondary school, academic secondary school), where they remain for up to 13 years of schooling (Van Ackeren and Klemm, 2011). PE is a compulsory subject in all school years and among all school types. In elementary schools, PE is coeducational and, according to the curricula, comprises three 45-min lessons weekly (Kulturministerkonferenz, 2017). Secondary school PE is either mono- or coeducational, depending on the federal state's regulations and comprises two to three 45-min sessions. The individual federal states' PE curricula mostly base on a concept of educative instruction, which justifies the importance of PE as a school subject by a double assignment (Neumann, 2004): Skill provision for self-determined lifelong participation in sports and other fields of movement cultures (movement education) and personal development through movement, games, and sports (general education). To accomplish this, most current German PE curricula follow a competence-oriented approach (Gogoll, 2013) as well as the didactic concept of *multiperspectivity* by Kurz (2004), emphasizing to teach movement, games, and sports in school PE by addressing six pedagogical perspectives (performance, expression, impression, health, cooperation, and risk).

Concerning time variables in PE, physical activities with a long involvement of time-on-task are considered as key conditions of sports- and movement-cultural competence acquisition and recognized as normative criteria of high-quality PE instruction (Stegemann, 2003; Gebken, 2005). In contrast to this theoretical meaning, there are only few educational studies on the movement time of students in PE in Germany (e.g., Kretschmer, 1970; Hoppe and Vogt, 1979; Hoffmann, 2011). Existing studies are further mainly observational and usually cover one low-achieving and/or one high-performing student per PE lesson with the help of a stopwatch and observation sheets. The results show an average movement time between 16.2 (Kretschmer, 1970) and 26.3% (Hoffmann, 2011) of the net PE lesson time. Overall, however, the majority of these studies are far behind current curricular developments and obtain limited validity due to small sample sizes per class and a large amount of research staff involved.

Within the *multiperspectivity* of German PE curricula, the pedagogical perspective “health” focuses on the health potential of PE and aims to promote students' health and develop health awareness (Kurz, 2004). However, the scientific involvement with the topic in Germany is mostly conceptually oriented (e.g., Balz, 1995; Brodtmann, 1998; Töpfer, 2017). Quantitative empirical data on health effects of German PE is sparse and particularly emphasizes direct health and fitness consequences of secondary school PE (e.g., Tittlbach et al., 2010; Höner and Demetriou, 2014). A key variable of most studies is movement intensity, typically measured by heart rate monitor (Adler et al., 2006; Fröhlich et al., 2008; Wydra, 2009), pedometer (Uhlenbrock et al., 2008), and accelerometer (Kobel et al., 2015, 2017; Kretschmann et al., 2016). Only two studies in Germany so far deal explicitly with MVPA in PE. Kobel et al. (2015) explored the amount and intensity of elementary school children's PA during PE lessons in southwest Germany via accelerometers. Students on average spent 8.5 min of each 45-min PE lesson in MVPA, favoring single-lesson PE periods over double-lesson PE periods. Boys were significantly more active than girls. Kretschmann et al. (2016) analyzed accelerometer data from an academic secondary school in Baden-Wuerttemberg (Germany). The students engaged in MVPA for 58.98% of their PE time available. Girls were more active than boys. Middle and upper grade levels showed significantly higher mean time in MVPA compared to lower grade levels.

### The Present Study

With the present study, we seek to expand international research on time and PA in PE with German PE data. Further, we aim to elaborate the so far marginalized research on PE time in Germany with continued data from the elementary school sector. For this purpose, we objectively quantify different degrees of intensity of PA and sedentary time in PE lessons and analyze them focusing on determinants that have an essential meaning for active living and health behaviors of the examined target group (Sallis et al., 2000, 2006; Trost, 2007). In particular, we focus on students' gender, grade, and weight status (Fairclough and Stratton, 2006; Hollis et al., 2016). We further consider entrenched correlates of children's PA and SB in Germany, i.e., voluntary sports club (VSC) membership, parents' sport status, and the general activity level (Schmidt et al., 2017; Demetriou et al., 2019), as well as the PE teachers' PE education status (Hollis et al., 2016).

## Methods

The present study is part of a larger research project aiming to explore PA in elementary schools in Baden-Wuerttemberg (Germany). Selected schools were full-day schools, providing either a mandatory or optional full-day school branch. The school organization form was selected in cooperation with the responsible *Ministry of Education and Cultural Affairs Baden-Wuerttemberg* (see Spengler et al., 2019). The study was approved by the Ethics Committee of the University of Konstanz, Germany (approval number: 08/2019), and was conducted in accordance with the Declaration of Helsinki.

### Participants

Eleven out of 25 relevant elementary schools (response rate: 44%) assented to participate in the research project. Out of these 11 schools, 1,620 students were invited to participate; 508 students (response rate: 31%) were equipped with accelerometers. The final sample included 328 children with compatible data on PA in PE lessons (47% male, mean age 8.7 ± 1.2 years). Dropouts were due to missing accelerometer data in the analyzed timeframe (*n* = 33) or inadequate wear time of accelerometer during PE lessons (*n* = 72, thereof *n* = 35 of one whole school in which PE involved swimming lessons in the investigation period). Fourteen additional cases had to be excluded due to technical errors (e.g., measured %SB > 90% during one 45-min PE lesson). Data collection took part from May to July 2017. Students of all elementary school grades, enrolled in either full-day or regular classes, were included in the sample. All parents and children gave verbal and written consent to the study. Information about class (e.g., students per grade, teachers PE education status) was given by the school principals or teaching staff. Sex, date of birth, height, and weight of the included students were assessed via self-report questionnaires. Body mass index (BMI = kg/m^2^) was calculated. Numbers of cases, means and standard deviations (SD) of age, height, weight, BMI, and the percentage of deviant weight status for boys and girls (Kromeyer-Hauschild et al., 2001) are presented in Table 1.

**Table 1:** Numbers of cases, means, and standard deviation of age, height, weight, BMI, and percentage of deviant weight statuses for boys and girls.

		***N* (range)**	**Age (years)**	**Height (in cm)**	**Weight (in kg)**	**BMI**		
						**(kg/m^**2**^)**	**UW (%)**	**OW (%)**
**Grade 1**								
Mean	Boys	29–42	7.28	127.27	26.25	16.06	17.2	13.8
(SD)			(0.45)	(5.90)	(5.73)	(2.98)	(38.4)	(35.1)
Mean	Girls	33–44	7.36	123.93	23.55	15.24	30.3	3.0
(SD)			(0.61)	(6.48)	(3.97)	(1.77)	(46.7)	(17.4)
**Grade 2**								
Mean	Boys	24–35	8.32	132.33	28.17	16.06	12.5	–
(SD)			(0.64)	(5.31)	(3.60)	(1.69)	(33.8)	–
Mean	Girls	42–57	8.27	131.18	28.28	16.38	16.7	11.9
(SD)			(0.49)	(6.08)	(6.26)	(2.60)	(37.7)	(32.8)
**Grade 3**								
Mean	Boys	41–47	9.43	139.32	34.50	17.46	12.2	24.4
(SD)			(0.54)	(7.37)	(8.709	(3.02)	(33.1)	(43.5)
Mean	Girls	36–41	9.41	136.39	30.68	16.40	8.3	5.6
(SD)			(0.55)	(7.85)	(5.78)	(1.86)	(28.0)	(23.2)
**Grade 4**								
Mean	Boys	26–28	10.29	146.42	40.07	18.44	–	19.2
(SD)			(0.46	(7.81)	(11.13)	(3.29)	–	(40.2)
Mean	Girls	22–31	10.21	143.65	35.78	17.33	18.2	9.1
(SD)			(0.49)	(7.51)	(6.93)	(2.46)	(39.5)	(29.4)
**Total**								
Mean	Boys	120–152	8.76	136.23	32.20	17.04	10.8	15.8
(SD)			(1.23)	(9.60)	(9.34)	(2.99)	(31.2)	(36.7)
Mean	Girls	133–173	8.64	132.78	29.15	16.27	18.0	7.5
(SD)			(1.15)	(9.43)	(7.03)	(2.28)	(38.6)	(26.5)

*N (range) denotes the range of cases for all categories per grade × gender. BMI categorization is based on Kromeyer-Hauschild et al. (2001) using the 10th and 90th percentile for underweight (UW) and overweight (OW)*.

### Measures

#### Physical Acitivty and Sedentary Behaviour

Participating students wore a tri-axial accelerometer (ActiGraph GT3X, GT3X+ or GT3XBT) at the left hip with an elastic belt. The wear period was set to seven consecutive days with different starting days and comprised a full school week for each student. Participants were asked to wear the accelerometer as soon as they got up in the morning and only remove it during water-based activities, at night, and in exceptional cases when there was a risk of injury (e.g., while performing contact sports such as martial arts). Periods of PE lessons were identified on the basis of participating classes' timetables. The test period was a regular school week without any special school sports or PE events. By wearing the accelerometer all the time, no PE time was lost due to putting it on and taking it off. PE teachers, children, and parents were blinded to the target of measuring PA in PE. Students had on average 1.71 ± 1.41 PE lessons per week, with 140 students participating in one PE lesson, 143 students experiencing two PE lessons, and 45 students taking part in three PE lessons per week (see Supplementary Materials for further and detailed information). Data collection by accelerometer was set to 10-s epoch length and started 1 to 2 h after distribution to avoid the recording of increased PA because of curiosity. We used ActiLife Software v6.11.9 for data processing. Wear Time Validation was calculated using Troiano 2007 defaults (Troiano et al., 2008); hence, time intervals of at least 60 consecutive minutes of zero counts were defined as non-wear time. Counts per minutes of acceleration from the vertical axis only were classified as sedentary behavior (SB; 0–99), light PA (LPA; 100–2,240), moderate PA (MPA; 2,241–3,840), and vigorous PA (VPA; 3,841–∞) according to Pulsford's validated cut points for children (Pulsford et al., 2011). Only students with a valid recording of at least one full PE lesson were included in the sample. Because of the different number of net PE lessons (i.e., incomparable number of PE minutes per student), PA levels in our study are relative values (%).

#### PA Behavior Correlates

To collect selected data on students' PA behavior, participants were asked to complete a paper–pencil survey together with their parents (including custodians). General PA, VSC membership, and status of sports within the family were assessed using scales from the MoMo PA questionnaire (Schmidt et al., 2016). The scales were checked for reliability and validity for children and adolescents aged 9–17 years (Jekauc et al., 2013b). The applied scales reflect an optimized version with slight modifications for the second wave of the MoMo Study and are also recommended for the target group in our study because of the lack of validated survey instruments (Schmidt et al., 2016). Regarding the general level of PA, we used a single item (“In a normal week, on how many days are you at least 60 min physically active?”) to be answered on a scale ranging from 0 to 7 days. For presentational purposes, this scale was collapsed to three categories indicating low (0–2 days per week), medium (3–5 days), and high (6–7 days) general activity. Applying a different operationalization with more categories did not show substantial differences but resulted in very small categories with large standard errors. Affiliation in VSCs was measured by asking participants if they are a member of at least one VSC. Regarding PA's relevance within the student's family, we used a combined item indicating whether at least one parent physically exercised on a regular basis (“Does your mum/your dad do sports regularly?”).

#### Teacher PE Education Status

German elementary school PE is widely taught by non-specialist teachers (Liebl and Sygusch, 2020). Regarding the PE education status of the teachers teaching PE lessons in the investigated classes, we differentiated between classes held by specialist PE teachers, classes held by non-specialist teachers, and classes held by teams of specialist and non-specialist teachers.

### Data Analyses

All analyses were conducted using STATA 14.1. We explored possible influencing factors on SB (%SB), LPA (%LPA), and MVPA (%MVPA) according to the choosen cut points (see Pulsford et al., 2011). For this purpose, we used comparisons of means (*t*-tests), applying a Bonferroni correction that accounts for multiple comparisons by adjusting the comparisonwise error rate. We tested for significant differences in the proportions of PA intensity levels during PE lessons that have been recorded by accelerometers regarding established PA and SB determinants of elementary school students (gender, grade, BMI, VSC membership, parents sport status, general activity level) as well as teachers' PE education status. Because most of these variables have not been used before as determinants of PA levels during PE lessons, our analyses are largely explanatory.

## Results

**Figure 1:**
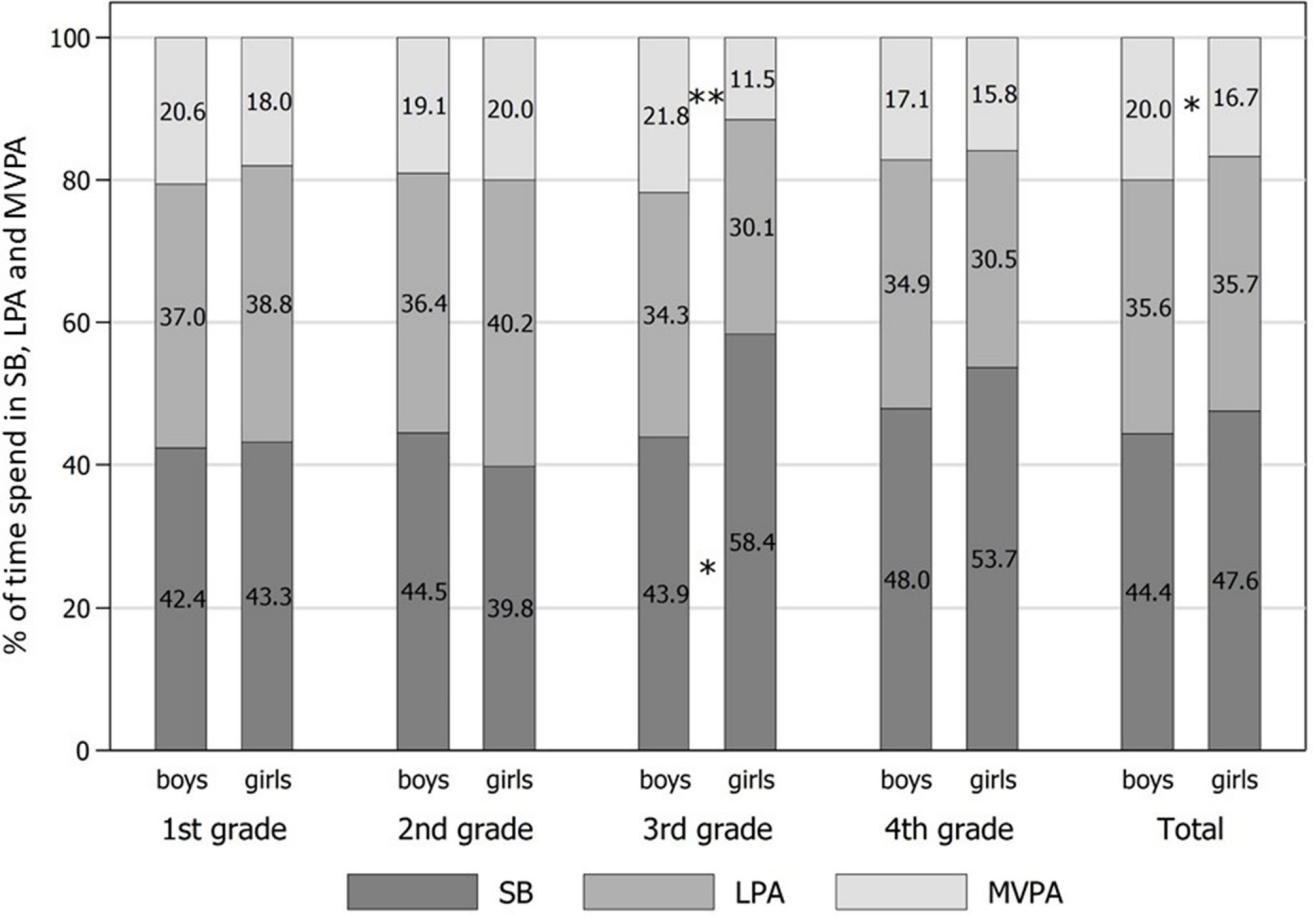
Levels of physical activity (PA) and sedentary behaviour (SB) by grade and gender of elementary school children. Entries are % means of sedentary behavior (SB), light physical activity (LPA), and moderate-to-vigorous physical activity (MVPA). Test of significant differences: *t* tests (two-sided). Significance level: *p < 0.05; **p < 0.01.

Figure 1 presents mean values of SB and PA levels in different school grades, differentiated by gender. Overall, girls show significant lower levels of %MVPA compared to boys (about −3% points, Cohen's *d* = 0.27). This result is mainly driven by differences in grade 3 (−10% points). In addition, the second finding is the much higher %SB of girls in grade 3. While girls spend 58% of PE in SB, boys in grade 3 show 44% of such behavior. Regarding girls, there is a clear gap between grades 1 and 2 on the one side and grades 3 and 4 on the other side, with the latter grades indicating higher %SB (40–43 vs. 54–58%) and lower %LPA (39–40 vs. 30–31%) during PE lessons. For boys the pattern is less clear.

Table 2 highlights %SB and levels of %PA of male and female elementary school students in relation to further indicators of PA behavior. In contrast to Figure 1, we only distinguish between boys and girls in order to have a statistically sufficient number of cases for comparison. With respect to BMI, we see that overweight girls have a lower share of %SB compared to girls showing normal BMI (−13% points). Overweight girls' share of %MVPA during the measured PE lessons is also higher (+8%-points). Underweight girls show a similar pattern (i.e., lower %SB and higher %MVPA compared to girls with a normal BMI). For boys, the differences are much smaller and far from being significant.

**Table 2:** Levels of physical activity (PA) and sedentary behaviour (SB) by key characteristics of elementary school children.

		**BMI**			**Membership in sports club**		**General activity**			**Parents doing sport**		**Teacher with PE study**		
		**Normal**	**UW**	**OW**	**No**	**Yes**	**Low**	**Med**	**High**	**No**	**Yes**	**No**	**Yes**	**Teams**
Boys	SB	44.3	47.7	42.5	44.9	44.6	46.6	43.1	45.3	45.7	43.7	38.9	44.3	50.2
	(SD)	(21.1)	(20.9)	(24.8)	(19.7)	(21.1)	(22.4)	(20.0)	(21.2)	(22.7)	(19.6)	(18.1)	(22.9)	(22.2)
	LPA	35.3	30.2	38.0	35.4	35.6	33.7	36.0	36.0	34.6	36.2	41.1	34.5^*^	31.5^**^
	(SD)	(11.6)	(7.5)	(11.6)	(10.8)	(11.2)	(11.6)	(10.9)	(11.1)	(12.1)	(10.4)	(10.8)	(10.8)	(11.7)
	MVPA	20.4	22.2	19.5	19.7	19.8	19.7	20.9	18.7	19.7	20.2	20.0	21.3	18.3
	(SD)	(13.2)	(16.1)	(15.9)	(14.0)	(12.8)	(15.3)	(12.9)	(13.3)	(13.7)	(13.0)	(11.5)	(15.0)	(13.6)
	N	88	13	19	31	114	16	68	59	54	98	30	74	25
Girls	SB	49.4	43.6	36.1	45.4	48.1	45.0	48.3	48.7	52.0	45.3^*^	42.9	48.3	51.6
	(SD)	(19.4)	20.0	(12.7)	(19.0)	(19.9)	(17.0)	(20.3)	(20.2)	(20.9)	(18.5)	(17.1)	(21.9)	(17.5)
	LPA	35.1	36.1	40.5	36.2	35.8	38.3	34.1	35.9	33.2	37.0^*^	40.7	34.0^**^	33.4^*^
	(SD)	(10.8)	(11.1)	(6.8)	(11.6)	(10.9)	(9.9)	(11.5)	(11.3)	(11.5)	(10.7)	(10.9)	(11.4)	(10.5)
	MVPA	15.5	20.3	23.4	18.4	16.2	16.7	17.6	15.4	14.8	17.7	16.4	17.7	15.1
	(SD)	(10.9)	(13.0)	(8.4)	(10.5)	(12.2)	(9.9)	(12.7)	(11.1)	(11.3)	(11.6)	(9.6)	(13.5)	(9.9)
	N	99	24	10	50	116	25	71	67	58	115	41	91	21

Entries are mean percentages of time spend in sedentary behaviour (SB), light physical activity (LPA), and moderate-to-vigorous physical activity (MVPA) with standard deviations (SD). Test of significant differences, t tests (two-sided; reference, first column of respective characteristic). Significance level:

* *p < 0.05*;

** *p < 0.01*.

*BMI categorization is based on Kromeyer-Hauschild et al. (2001) using the 10th and 90th percentile for underweight (UW) and overweight (OW). General activity refers to physical activity (PA) of at least 60 min per day in a normal week, divided into low (0–2 days per week), medium (3–5 days), and high (6–7 days)*.

With respect to membership in VSC, there are no significant differences. However, the general tendency points in the direction that VSC membership for girls is associated with a higher share of %SB (+3% points) and lower %MVPA (−2% points). For boys, the differences are negligible.

Regarding general activity, boys stating higher general activity show a lower share of %SB (−3% points for medium and −1% points for high general activity) and a slightly higher share of %LPA (+2% points for medium/high general activity) than boys reporting low general activity. The opposite tendency is visible for girls. Girls who stated to be physically active for at least 3 days show a slightly lower share of %LPA (−4% points for medium and −2% points for high general activity) and a higher share of %SB (+3% points for medium and +4% points for high general activity) compared to girls reporting low levels of general activity. However, these differences are not significant at the 95% level.

The previous pattern of generally larger differences with respect to girls continues for the status of sports within the family. Girls with at least one parent doing sports on a regular basis show a significantly lower share of %SB (−7% points, Cohen's *d* = 0.35) as well as a significant higher share of %LPA (+4% points, Cohen's *d* = 0.35). For boys, the parental background seems to play a less important role, although the effects are similar, i.e., boys show less SB and are more physically active during PE lessons when at least one parent exercises regularly.

Finally, we looked at the PE education status of the teachers teaching the examined PE classes. Here, we distinguished between children in PE lessons guided by specialist (*n* = 177) and non-specialist PE teachers (*n* = 72) and lessons taught by teams consisting of specialist and non-specialist PE teachers (*n* = 48). PE lessons guided by specialist PE teachers or teams with at least one teacher having a PE degree are characterized by a significantly lower %LPA for both boys (−7 to −10% points, respectively; Cohen's *d* = 0.62–0.86) and girls (about −7% points, Cohen's *d* =0.60–0.68). This result is supplemented with the (not significant) finding that both boys (+11% points) and girls (+9% points) show a higher %SB during PE lessons if at least one teacher is a PE specialist.

## Discussion

The present research takes up PE's possibility to contribute substantially to individual and public health by ensuring sufficient levels of PA. Specifically, we addressed selected personal and environmental determinants of students' PA levels and SB in German elementary school PE lessons. Overall, girls and boys are on average 17 and 20% of their PE time active in MVPA, which falls rather short in comparison to the 50% recommended as health contributing by national and international references (U.S. Department of Health and Human Services, 2000; Rütten and Pfeiffer, 2016). Pronounced phases of PE time in SB additionally support the rather limited health-related activity level of the analyzed PE lessons.

In sum, our results show only few significant variations. This is partly due to the rather small overall sample size and the even smaller group sizes of certain determinants. However, it is apparent that students' gender and grade largely moderate the detected differences. Firstly, girls show significantly lower levels of MVPA and tend to have higher levels of SB compared to boys. Secondly, with increasing school age, the percentages in MVPA and SB levels both develop negatively from a health perspective—even greater in girls. Regarding gender, the results thus confirm a series of international findings where girls obtain lower levels of mean MVPA and higher levels of mean SB in elementary school PE (Meyer et al., 2013; Fröberg et al., 2017; Kirkham-King et al., 2017; Tanaka et al., 2018; Zimmo et al., 2020). However, there are also studies that do not report significant gender differences (Verstraete et al., 2007) or tendentially higher percentages of MVPA in elementary school PE for girls (Nettlefold et al., 2011). Regarding grade, our data show a similar pattern as for gender. The results are in line with a previous study, which linked lower grades with significant more time in MVPA and significant less time in SB in comparison to older grades (Tanaka et al., 2018). At the same time, other studies report a significant rise of MVPA per elementary grade (Kirkham-King et al., 2017) or similar PA levels across grades (Meyer et al., 2013; Zimmo et al., 2020).

The detected gender differences stabilize in the relationship of weight status and mean PE time spent in PA and SB. In line with findings that did not determine a statistically relevant connection between BMI and PA for PE time (Meyer et al., 2013; Spessato et al., 2013; Kobel et al., 2017; Zimmo et al., 2020), our study shows a tendentially health-promoting difference for overweight and underweight girls. For both overweight and underweight girls, weight status similarly showed connections to less PE time spent in SB and more PE time spent in MVPA compared to girls with a normal weight status. Given the fact that overweight and obesity are negatively associated with various health factors, PE lessons here seem to depict a suitable setting for health-promoting PA levels for these groups. For boys, there are no statistically relevant differences in PA level intensities regarding weight status.

To further understand PA and SB levels, we queried established correlates of active living and health behaviors. Data regarding memberships in VSCs and general activity levels, i.e., indicators that point to a physically active time outside school and a specific affinity to PA, further stabilize the different findings regarding gender. While the differences among boys are again either negligible (VSC membership) or according to expectation (general activity), girls' results are counterintuitive. With this, the picture of PE lessons that at best are partially appealing for girls becomes clearer. This carries further weight, considering that girls obtain high degrees of involvement in voluntary organized sports, which is furthermore a characteristic for girls in Germany in general (Jekauc et al., 2013a).

The comparison of mean PA and SB levels in PE with PA status within the students' families showed expected tendencies (i.e., less SB and higher PA in PE for students with physically active parents). The results thus confirm the general meaning of parents as social role models for children's physical activities and underline previous research emphasizing physically active and sportive parents as primary agents for the physical and sporting socialization of their children (Pugliese and Tinsley, 2007; Timperio et al., 2013) for the so far minor research field of PE. The apparent stability of parental impact across PA settings is particularly interesting against the background of diverging social logics (guiding actions within the settings). In contrast to distinct social functions of PA outside and inside school (i.e., communication of physical achievements vs. educational goals; see Stichweh, 2013), children seem to be influenced by their parents regardless of the setting and adapt PE as an arbitrary movement offer among others. Further, as our comparison so far bases on a generally formulated single item indicating whether at least one parent physically exercised on a regular basis, the study does not yet allow for differentiated explanations. Here, taking into account the positive correlations between parental PA being operated together with their children, parental support of children's PA, and child PA (Trost and Loprinzi, 2011) could prove to be a worthwhile approach for deepening the variable and should be considered in future studies.

In contrast to a number of studies comparing PA levels of PE lessons taught by specialist and non-specialist PE teachers (Faucette et al., 1990; McKenzie et al., 1995, 2001; Telford et al., 2013), the present study attributes PE lessons held by non-specialists comparatively enhanced health-promoting PA levels. This is surprising at first but may gain plausibility due to different reasons. First, the existing findings all arise from intervention studies explicitly geared toward fitness and health-promoting PA in PE and carried out in countries in which PE and thus University education of PE teachers focus increasingly on health. The internationally different design of PE curricula offers a second explanation. PE curricula in the German federal states are explicitly aligned in a multi-perspective manner and fix health as one of several possible pedagogical perspectives according to which PE can be designed (Kurz, 2004). In some of the observed PE lessons, specialist PE teachers possibly may have pursued goals different to PA and health, while the non-specialist PE teachers may have (unwittingly) used the lessons as PA favoring playtime. Third, it might be possible that the specialist PE teachers are either (still) stuck in outmoded didactical PE concepts (e.g., *concept of traditional sports*, Söll, 2000) that find little access in motivating the current generation of students for PA or the PA periods are constrained by orientations on current sport didactical concepts (e.g., *reflective agency in movement, games and sports*, (Schierz and Thiele, 2013) *sport-cultural and movement-cultural competence*, Gogoll, 2013) that explicitly implement cognitive learning and reflection phases in PE. Further analyses are needed to explain the differences. In our opinion, worthwhile starting points might be associations between PA levels in PE and the teachers' understanding of sport and PE (e.g., leading PE didactical models, teaching styles) on the one hand and facets of content, structure, and design of PE lessons (e.g., curricular aims, types of physical activities, teaching methods, lesson periods) on the other hand.

Besides several major strengths of the study (especially device-based measured PA levels, a comparatively large sample size, high ecological validity, and integration of PA behavior variables), there are some limitations to be considered when interpreting the data. Key detriments of the study were the low generalizable sample (i.e., schools from only one German federal state, voluntary school participation, no random design), self-reported data for height and weight, lack of personal and contextual information (e.g., teacher's understanding of PE, content of PE lessons), and the cross-sectional design that does not allow for statements of causal relationships and trajectories.

## Conclusion

The main purpose of this study was to objectively quantify different levels of PA and SB in German elementary PE lessons and analyze them focusing on selected determinants of active living and health behaviors. The analysis of PA levels and sedentary time revealed generally insufficient exercise times as well as gender and grade differences highlighting older girls as the less active group. Important determinants of PA levels and sedentary time in PE were deviant weight status and physically active parents for girls as well as PE lessons taught by at least one specialist PE teacher for boys and girls. If one takes the requirements and possibilities of health promotion by and through PE seriously, it therefore seems necessary to re-design elementary PE lessons considering personal und environmental influencing variables regarding active living and health behaviors. Because of a preciously little chance of contextual changes that raise PA in (German) PE (e.g., increases in PE class frequency or duration, reduction of PE class size), due to education politics' regulations, it is essential to use existing PE structures more effectively and more target group oriented. This requires an adequate focus and skills (i.e., subject-didactic, organizational, and pedagogic-psychological competencies) of PE teachers. In the course of this, the abovementioned multi-perspective curricular alignment of German school PE (Kurz, 2004) and the school's mandatory educational “refraction” of health promotion, however, must not fall from view, if PE is to remain an essential but independent health factor of modern society (Kirk, 2006; Cale et al., 2014).

## Data Availability Statement

The datasets for this study can be found in the Supplementary Material.

## Ethics Statement

The studies involving human participants were reviewed and approved by Ethics committee of the University of Konstanz, Germany (approval number: 08/2019). Written informed consent to participate in this study was provided by the participants' legal guardian/next of kin.

## Author Contributions

AK and FM conceived and designed the overall study. AK collected data. DJ designed and organized the sub-study of this paper. AK and MB performed the data preparation. MB performed the statistical analyses. DJ wrote most of the paper with substantial contributions from MB, CM, and FM. All authors provided feedback on drafts and approved the final manuscript.

## Conflict of Interest

The authors declare that the research was conducted in the absence of any commercial or financial relationships that could be construed as a potential conflict of interest.
